# Effects of an innovative educational program on female athletes' knowledge and habits related to pelvic floor care: a quasi-experimental study

**DOI:** 10.3389/fpubh.2025.1664696

**Published:** 2025-09-05

**Authors:** Elena Vico-Moreno, Elisa Bosch-Donate, Juan Carlos Fernández-Domínguez, Andreu Sastre-Munar, Beatriz Bachero-Mena, Natalia Romero-Franco

**Affiliations:** ^1^Nursing and Physiotherapy Department, University of the Balearic Islands, Palma de Mallorca, Spain; ^2^Health Research Institute of the Balearic Islands (IdISBa), Palma de Mallorca, Spain; ^3^Department of Human Movement and Sport Performance, University of Seville, Seville, Spain

**Keywords:** female, pelvic floor disorders, education, distance, sports, habits

## Abstract

The physical demands of sports require female athletes to be aware of pelvic floor (PF) care. However, knowledge about PF dysfunctions and unhealthy habits remains insufficient in sports populations. Innovative and specific educational strategies are needed to engage female athletes. This study evaluated the effects of an online educational program on PF knowledge and care habits in female athletes. A three-session online program with practical content and 3D models was implemented. Of the 130 registered athletes (35.9 ± 11.2 years old), 70 fully attended (FA), 23 partially attended (PA), and 37 did not attend (NA). Before and 4 weeks after the program, participants completed a questionnaire assessing: (1) demographics and sports data; (2) PF symptomatology; (3) knowledge of urinary incontinence (UI), female sexual dysfunction (FSD), ano-rectal incontinence (ARI), and pelvic organ prolapse (POP); and (4) habits during micturition, defecation, and sports practice. FA and PA athletes rated the utility of the program (0–10 scale). After the program, FA athletes showed improved knowledge of UI (*p* = 0.032), FSD, ARI, and POP (*p*_s_ < 0.001) compared to baseline and NA athletes (*p*_s_ < 0.001). FA athletes also reduced unhealthy habits during micturition (*p* = 0.003) and defecation (*p* = 0.003), and had fewer unhealthy habits overall compared to NA athletes. The utility of the program was rated 8.4 points. A three-session innovative online educational program proved effective in enhancing PF-related knowledge and improving care habits among female athletes.

## 1 Introduction

In sports, the high diary physical demands require females to be aware of pelvic floor (PF) care to achieve maximum performance with a functional and healthy PF (1). Despite the abundance of available information on the internet, the quality of health-related resources is not always ensured owing to the normalized or silenced nature of discussions regarding PF disorders (2). In sports, this silence is often reinforced by gender stereotypes and cultural taboos that discourage open conversations about pelvic health, especially among younger athletes (3). Consequently, many female athletes report feeling embarrassed or ashamed to disclose PF symptomatology (4).

Even today, knowledge related to the PF function is insufficient among sports populations (3, 5). Consequently, females are unconscious of their unhealthy toileting habits or behaviors during sports practice (5, 6). Some habits that increase intra-abdominal pressure, such as holding breath during intense exercise or forgetting to activate the PF musculature during exercises, are common among athletes. As these habits are related to PF symptomatology, it is necessary to urge athletes to reduce these habits (7). As a recent review has showed, those athletes with poor knowledge of PF have often detrimental habits for the PF care like absence of health-seeking care or low engagement of pelvic floor muscle training (PFMT) (8, 9). A comprehensive cross-sectional study at the Lima 2024 World Athletics U20 Championships revealed that over 50% of elite female youth athletes reported PF symptoms, yet fewer than 30% were aware of PF health and only 12% had ever undergone screening (9). Instead, athletes often acquire coping strategies to avoid symptoms, such as limiting the social and sports contexts. These practices end up affecting their quality of life and sports performance (10, 11).

As sports populations often prioritize maximum performance over PF health, it is important to design specific educational strategies that focus on sports-related aspects. This approach will help athletes acquire PF knowledge and reduce unhealthy habits. In this regard, new technologies can aid in designing attractive educational formats that increase athletes' participation, even in those without PF disorders. Among innovative resources, virtual practical content can engage participants and increase their awareness of their PF structures (12). When educational interventions incorporate practical skills to locate and correctly contract PF, females often improve the proprioception and contractile capability of these structures (13). Additionally, dynamic and interactive 3D models could support anatomical explanations and practical components, making easier for younger females to understand these complex structures (14).

Although several educational programs have been developed to address PF in athletes, most are designed for clinical populations and lack sport-specific adaptations (15, 16). These educational actions often used exclusively theoretical information (17), or video-based non-interactive presentations (14, 18, 19). These resources may not motivate the participation of asymptomatic and/or younger female athletes.

Therefore, this study aimed to evaluate the effectiveness of a three-session innovative online educational program on PF care, based on changes in female athletes' knowledge and habits related to its maintenance. We hypothesized that athletes who attended all sessions of the educational program would increase their knowledge of all PF disorders. Because behavioral change requires time, we hypothesized that we would at least observe a trend toward improved habits related to PF care in athletes who attended the entire educational program.

## 2 Material and methods

### 2.1 Design

A quasi-experimental pre-test-post-test design with non-equivalent groups was conducted. This type of design is used when random assignment is not feasible, allowing for the evaluation of intervention effects in real-world settings (20). During April and May 2024, females who practiced sports in Spain were invited to participate in a 3-week online educational program on PF function and care. The invitation was sent via e-mail throughout their sports federation, teams or training groups, and announced in social networks (Instagram^®^ and X^®^). Before and 4 weeks after the intervention period, females responded to an online questionnaire to assess their knowledge of PF function and habits related to their care. Sociodemographic, sports-related, and PF symptomatology data were collected before the start of the study. Due to the anonymous nature of the questionnaire, the athletes were asked to add a personal code that could be remembered and recovered independently. This personal code made it possible to connect initial and final responses and assess potential changes. Participants were not assigned to groups but rather self-selected based on their engagement with the educational program. All female athletes were invited to complete the pre- and post-intervention questionnaires, regardless of their participation in the sessions. As a result, three naturally formed groups emerged: full attendance—FA—those who attended all sessions; partial attendance—PA—those who attended partially; and non-attendance—NA—those who did not attend but completed both questionnaires. This allowed for comparative analysis of changes in knowledge and habits across varying levels of engagement to the educational program. Female athletes received feedback on their results and appropriate responses only after completing the post-intervention questionnaire.

As the National Sports Council certified ~740.000 sports licenses for female athletes aged at least 16 years in Spain in 2023, the sample size was calculated by considering a finite population. With a 95% confidence level and 80% statistical power, a minimum of 87 females was needed to detect a 15% mean difference and a 0.2 standard deviation in knowledge related to female sexual dysfunctions (FSD), which is identified as the lowest domain of knowledge that previous studies recommend improving (3).

**Figure 1:**
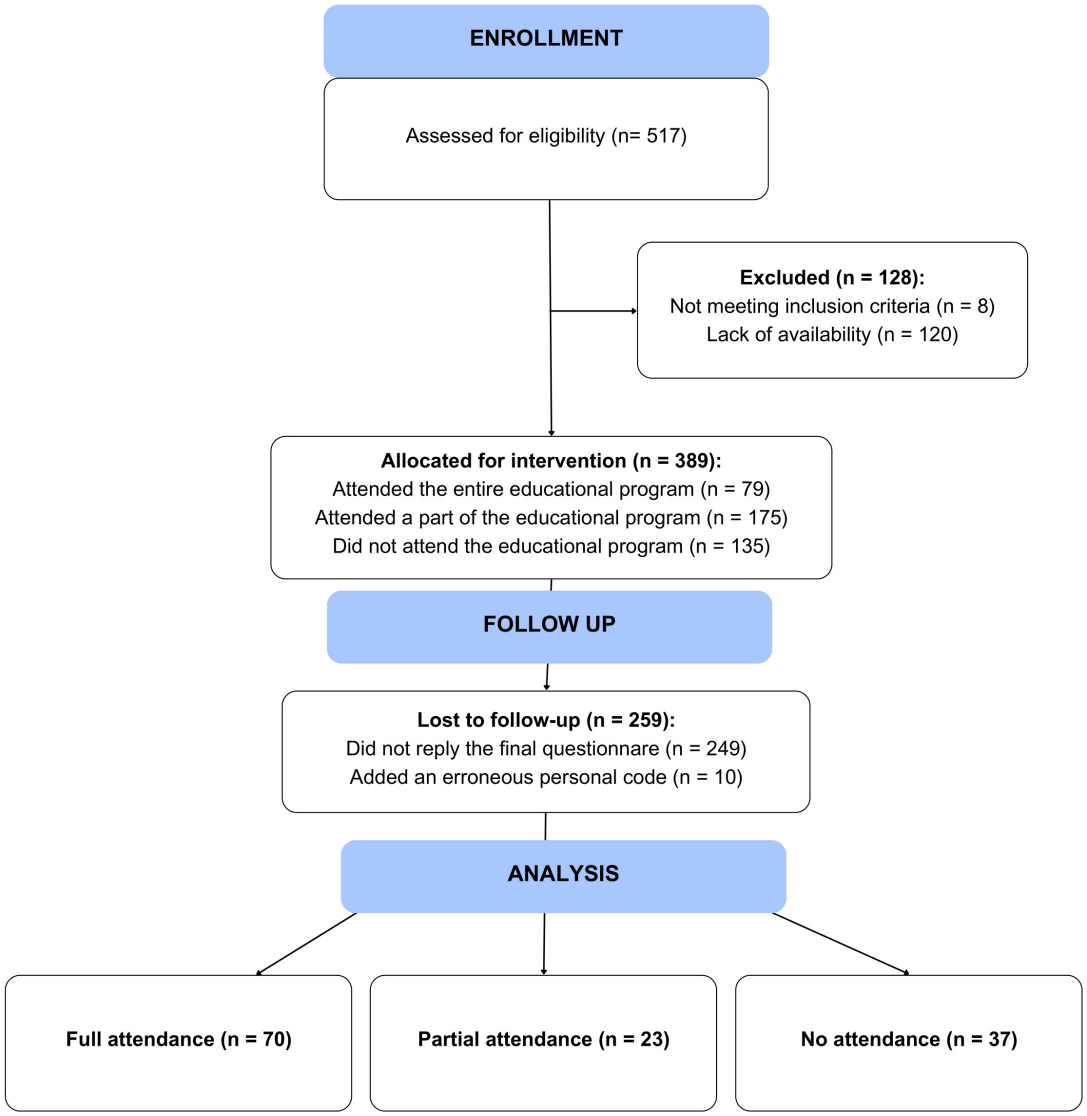
Flow diagram.

To participate in the study, females were required to train and compete in any sports modality in Spain, be 16 years old or older, and be able to understand Spanish instructions to complete the anonymous questionnaire. A total of 140 females responded to the initial and final questionnaires. However, 10 participants were excluded from the statistical analysis because an error in their personal code made it impossible to link the initial and final responses to the questionnaire (Figure 1).

Before enrolment, all females were informed about the study and provided written informed consent. This study was approved by the Ethical Committee of the University of the Balearic Islands (124CER19) and registered at clinicaltrial.gov (NCT06355297).

### 2.2 Online questionnaire

The questionnaire was created using the Jotform platform (San Francisco, CA, USA) and distributed through a sharing link. This questionnaire was designed by considering previous similar studies (3) and was created in two versions: the initial questionnaire (before the educational intervention period) and the final questionnaire (4 weeks after the educational intervention period). For the initial version, the following data were collected: (1) sociodemographic information (age and educational level), (2) sports data (years of experience and sport); (3) symptomatology related to PF function, including urinary incontinence (UI) based on ICIQ-SF (International Consultation on Incontinence Questionnaire—Short Form) (21); ano-rectal incontinence (ARI) based on responses to the Wexner questionnaire (22); dyspareunia, defined as pain just before, during or after sexual relationships (23); and pelvic organ prolapse (POP), identified by a bulging sensation in the vagina (24); (4) level of knowledge related to UI, ARI, POP, or FSD; and (5) habits related to PF care, such as micturition, defecation, and sport training (25, 26).

The level of knowledge related to UI and POP was compounded by 12 and 8 items, respectively, selected from Prolapse and Incontinence Knowledge Questionnaire, validated in Spanish (27) while ARI and FSD knowledge were compounded by 10 items extracted from similar previous studies (3). The level of knowledge was considered adequate when 70% of the responses were correct (28). The habits section was dichotomized based on whether a female performed the behavior or not: “yes” = sometimes, frequently, or always; and “no” = never or rarely. A behavior is considered a habit when it is carried out regular or with consistent practice (29). For each habits section, affirmative responses regarding unhealthy habits were summed to calculate a score. Each unhealthy habit, defined as detrimental to PF health, was assigned one point, following approach used in previous studies (29).

This questionnaire incorporated validated scales in Spanish for use with female populations. Specifically, for the assessment of PF symptoms, we used the ICIQ-SF (21) and the Wexner scale for bowel dysfunction (22, 30), both of which have demonstrated adequate psychometric properties in previous studies involving Spanish-speaking females, with Cronbach's alpha of 0.74 and 0.93, respectively. Additional items were included based on the official definition of dyspareunia and POP. For the evaluation of knowledge related to UI and POP, key items were selected from PIKQ, which has shown a Cronbach's alpha of Aydemi et al. (31). Items assessing knowledge of ARI and FSD were adapted from a previous study that reported good psychometric properties, with Cronbach's alpha values of 0.75 and 0.70, respectively (3). Regarding habits, those related to micturition and defecation were extracted from the Toileting Behavior (TB) scale, which has a Cronbach's alpha of 0.85. Due to the lack of validated tools specifically designed to assess sports-related PF care habits, the team of experts developed new *ad-hoc* items. Previously, the face validity and content validity of this section was verified by requesting collaboration of three experts (three pelvic floor therapists), and one independent expert (a general practitioner). Also, 15 female athletes fulfilled the questionnaire to confirm the appropriateness of the *ad-hoc* items (32). For validity, open fields options were included in each section to allow suggestions or comments related to adequacy and relevance criteria (content validity), as well as understanding and clarity (face validity). Internal consistency of *ad-hoc* items was supported with a Cronbach's alpha of 0.77.

Four weeks after the intervention period, the participants responded to the final version of the questionnaire. This final version was similar to the initial one: the 1st and 2nd sections were deleted, and a new section related to the educational intervention was added: attention to the workshop (full: if they attended all the sessions; partial: if they attended at least one session; or none: if they did not attend any session), and the perceived utility of the educational program for learning about PF care [rated from 1 (not useful) to 10 (essential)], applicable only to those who attended the program either fully or partially.

### 2.3 Educational program

The online educational program lasted 3 weeks, with one 60-min session per week, scheduled seven days apart. The structure of each session was as follows: a general introduction to the session content (10 min), practical exercises focused on PF proprioception (20 min), theoretical content on PF anatomy and PF (20 min), and a closing segment with take-home messages, debriefing, and Q&A (question and answer) (10 min) (33). The practical component included proprioceptive exercises led by a specialist physiotherapist, aimed at enhancing athletes' awareness of the osseous, muscular, and visceral structures of the PF. The theoretical contents was delivered using innovative resources such as a custom-designed 3D model accessible online (accessed at https://actitudproject.es/wp-content/uploads/2023/05/index.htm) to facilitate anatomical understanding. The sessions were organized as follows: Session 1 covered PF anatomy and physiology; Session 2 addressed PF function and dysfunction; and Session 3 focused on risk factors for PF disorders and strategies for PF care. The structure was designed based on previous studies (8), combining theoretical content with practical exercises to enhance attentional processes and learning outcomes (34); incorporating verbal instructions to guide participants in perceiving and contracting PF muscles, thereby improving proprioception (13); and integrating interactive and visual 3D formats to support comprehension and engagement in PF education for female athletes (35). Given that feelings of shame associated with PF disorders may inhibit participants from asking questions openly, an anonymous online tool was provided to allow athletes to submit questions confidentially. Addressing these questions during the final segment of each session likely contributed to increased engagement and retention throughout the program.

### 2.4 Statistical analysis

Mean and standard deviation (SD) and frequencies are reported for numerical and categorical variables, respectively. The participants were divided into three groups according to their attendance in the educational program (full attendance, FA, Partial attendance, PA, No-attendance, NA). As the data were not normally distributed for any continuous variable, the baseline characteristics of the participants were compared using the Kruskal–Wallis test. This test was also used to compare the level of knowledge and number of unhealthy habits of participants according to their group at post-intervention measurements. For statistical significance, the *post-hoc* Bonferroni Kruskal–Wallis test was used to explore the comparisons. The Wilcoxon test was employed to explore within-group differences. Confidence interval (CI) 95% were calculated for all differences, and effect sizes were obtained and interpreted using Cohen procedures, as follows: small (d ≤ 0.2), moderate (0.2 > d ≤ 0.8), or large (d > 0.8) (36). International Business Machines SPSS Statistics for Windows (version 24.0; Chicago, IL, 224 USA) was used, and statistical significance was set at *p* < 0.05.

## 3 Results

### 3.1 Characteristics of the participants

Of the 130 participants, 70 completely attended the educational program (FA), 23 partially attended the educational program (PA), and 37 did not attend the educational program (NA). Of all the participants, 86.9% had completed university studies, with 68.5% of them having pursued health-related fields (Table 1).

**Table 1:** Characteristics of participants.

**Characteristics**	**All participants (*n* = 130)**
Age (years old)^¥^	35.9 (11.2)
Sports experience (years)^¥^	12.3 (10.4)
Training volume (hs/week)^¥^	7.2 (5.4)
Vaginal delivery (%)	
No	56.8
Yes	32.2

¥, given as mean (standard deviation).

Regarding the prevalence of pelvic floor dysfunctions (PFD) among the participants, 47.1% had UI according to the ICIQ-SF, 37.1% had dyspareunia according to the key question, 20.0% had ARI according to the Wexner questionnaire, and 10.0% had POP according to the feeling of a bug.

Regarding the utility of the educational program according to the FA athletes' perception, it was rated with 8.41 ± 1.66 points, with 34.3% of athletes rating the program with 10 points, the highest score. The PA athletes gave an average score of 8.39 ± 1.56 points, with 36.0% of athletes giving the highest score.

### 3.2 Level of knowledge

The level of knowledge related to all domains before and 4 weeks after the intervention period is detailed in Table 2. No between-group differences were observed in the baseline measurements for any knowledge domain (*p* > 0.05). Statistically significant differences were found in the post-intervention measurements of knowledge related to UI (*p* = 0.001), FSD (*p* < 0.001), ARI (*p* < 0.001), and POP (*p* < 0.001). Between-group differences were found in post-intervention measurements for all domains (*p*_s_ < 0.001), with a lower level of knowledge for NA athletes compared to FA athletes in all domains (UI: *p* = 0.032; FSD: *p* < 0.001; ARI: *p* < 0.001; POP: *p* < 0.001), and compared to PA athletes regarding UI (*p* = 0.032) and POP (*p* = 0.018). No significant differences were observed between the PA and FA athletes (*p* > 0.05) (Table 2). Regarding within-group differences, only the FA athletes improved all levels of knowledge (UI: *p* = 0.002; FSD, ARI, and POP: *p* < 0.001), whereas the PA athletes improved only the level of knowledge related to UI (*p* = 0.021). No other within-group differences were observed (*p* > 0.05).

**Table 2:** Level of knowledge of participants, according to their attendance to the educational program.

**Knowledge domain**		**No-attendance group (NA) (*****n*** = **37)**		**Partial-attendance group (PA) (*****n*** = **23)**		**Full-attendance group (FA) (*****n*** = **70)**		**Between-group differences (at post)**			
		**Mean**	**SD**	**Mean**	**SD**	**Mean**	**SD**		**Mean dif**.	**95%CI**	**d Cohen**
**UI knowledge (score max: 12)**											
Pre-intervention		8.89	2.89	9.13	2.87	9.94	2.00	NA vs. PA	1.70^*^	(0.28–3.12)	1.17
Post-intervention		8.43	3.23	10.13	2.26	10.59	1.39	PA vs. FA	0.46	(−0.83–1.74)	NS
Within-group differences	Mean (95%CI)	−0.46	(−1.29–0.37)	1.00^*^	(0.02–2.02)	0.64^**^	(0.26–1.03)	NA vs. FA	2.15^**^	(1.06–3.24)	1.06
	d Cohen	NS		0.39		0.38					
**FSD knowledge (score max: 10)**											
Pre-intervention		5.03	2.63	5.61	2.81	6.20	2.41	NA vs. PA	1.47	(−0.08–3.03)	NS
Post-intervention		5.14	2.95	6.61	2.95	7.40	1.86	PA vs. FA	0.79	(−0.62–2.20)	NS
Within-group differences	Mean (95%CI)	0.11	(−0.51–0.73)	1.00	(−0.24–2.24)	1.20^***^	(0.75–1.65)	NA vs. FA	2.26^***^	(1.07–3.46)	1.01
	d Cohen	NS		NS		0.56					
**ARI knowledge (score max: 10)**											
Pre-intervention		6.92	1.99	7.17	2.17	7.71	1.79	NA vs. PA	1.19	(−0.02–2.13)	NS
Post-intervention		6.59	1.88	7.78	1.41	8.41	1.22	PA vs. FA	0.63	(−0.22–1.49)	NS
Within-group differences	Mean (95%CI)	−0.32	(−0.86–0.21)	0.61	(−0.07–1.28)	0.70^***^	(0.35–1.05)	NA vs. FA	1.82^***^	(1.10–2.54)	1.25
	d Cohen	NS		NS		0.47					
**POP knowledge (score max: 8)**											
Pre-intervention		5.31	2.33	6.09	2.00	6.01	1.92	NA vs. PA	1.54^*^	(0.42–2.65)	1.08
Post-intervention		5.03	2.35	6.57	1.56	6.76	1.35	PA vs. FA	0.19	(−0.81–1.19)	NS
Within-group differences	Mean (95%CI)	−0.28	(−0.78–0.23)	0.48	(−0.29–1.25)	0.74^***^	(0.34–1.14)	NA vs. FA	1.73^***^	(0.87–2.58)	1.02
	d Cohen	NS		NS		0.45					

ARI, ano-rectal incontinence; CI, confidence interval; FA, full-attendance group; NA, no-attendance group; NS, non-significant; PA, partial-attendance group; POP, pelvic organs prolapse; SD, standard deviation; UI, urinary incontinence; ^*^p < 0.05; ^**^p < 0.01; ^***^p < 0.001.

### 3.3 Habits

The number of unhealthy habits during sports practice, micturition, and defecation before and after the intervention are detailed in Table 3. When comparing pre-intervention measurements, significant differences were detected in unhealthy habits related to defecation (*p* = 0.031). At the post-intervention measurement, NA athletes had a greater number of unhealthy habits during sports practice (*p* = 0.036), micturition (*p* = 0.003), and defecation (*p* = 0.003) than those of FA athletes. Regarding within-group differences, only FA athletes had a reduced number of unhealthy habits during micturition (*p* = 0.050) and defecation (*p* = 0.026) compared to those of baseline. No other between-group or within-group differences were observed (*p* > 0.05). The occurrence of each habit is detailed in Supplementary Table 1.

**Table 3:** Number of unhealthy habits of participants, according to their attendance to the educational program.

**Habits**		**No-attendance group (NA) (*****n*** = **37)**		**Partial-attendance group (PA) (*****n*** = **23)**		**Full-attendance group (FA) (*****n*** = **70)**		**Between-group differences (at post)**			
		**Mean**	**SD**	**Mean**	**SD**	**Mean**	**SD**		**Mean dif**.	**95%CI**	**d Cohen**
**Sports unhealthy habits (** ***n*** **)**											
Pre-intervention		1.73	1.54	1.17	1.19	1.04	1.28	NA vs. PA	0.17	(−0.72–1.06)	NS
Post-intervention		1.86	1.44	1.70	1.61	1.17	1.26	PA vs. FA	0.52	(−0.28–1.33)	NS
Within-group differences	Mean (95%CI)	0.14	(−0.24–0.51)	0.52	(−0.12–1.16)	0.13	(−0.14–0.40)	NA vs. FA	0.69^*^	(0.02–1.37)	0.52
	d Cohen	NS		NS		NS					
**Urinary unhealthy habits (** ***n*** **)**											
Pre-intervention		2.73	2.04	2.70	1.64	2.01	1.92	NA vs. PA	0.94	(−0.33–2.22)	NS
Post-intervention		3.16	2.43	2.22	1.91	1.63	1.72	PA vs. FA	0.59	(−0.57–1.75)	NS
Within-group differences	Mean (95%CI)	0.43	(−0.15–1.02)	−0.48	(−1.26–0.30)	−0.39^*^	(−0.80 to −0.03)	NA vs. FA	1.53^**^	(0.56–2.51)	0.78
	d Cohen	NS		NS		−0.21					
**Fecal unhealthy habits (** ***n*** **)**											
Pre-intervention		2.59	1.71	1.65	1.34	1.86	1.53	NA vs. PA	0.44	(−0.67–1.56)	NS
Post-intervention		2.70	1.79	2.26	2.24	1.56	1.50	PA vs. FA	0.70	(0.21–1.71)	NS
Within-group differences	Mean (95%CI)	0.11	(−0.21–0.43)	0.61	(−0.16–1.37)	−0.30^*^	(−0.63 to −0.03)	NA vs. FA	1.15^**^	(0.29–2.00)	0.71
	d Cohen	NS		NS		−0.20					

ARI, ano-rectal incontinence; CI, confidence interval; FA, full-attendance group; NA, no-attendance group; NS, non-significant; PA, partial-attendance group; POP, pelvic organs prolapse; SD, standard deviation; UI, urinary incontinence; ^*^p < 0.05; ^**^p < 0.01.

## 4 Discussion

The main findings of this study demonstrate that a three-sessions online educational program with practical content and 3D resources was effective in increasing the level of knowledge related to all PFD domains. It was also useful in reducing unhealthy toileting habits among female athletes, although it was not sufficient for acquiring healthy habits during sports practice. These results were observed in FA athletes but not in PA athletes. Hence, the perception of athletes regarding the utility of the program was very high, with more than 30% rating educational interventions as essential.

Our results showed that 4 weeks after the educational program, athletes who attended all the three sessions improved their level of knowledge related to all PFD domains in comparison with baseline and athletes who did not attend any session of the educational program. These results are especially remarkable for the FSD domain, in which only females who attended the program completely reached an appropriate level of knowledge (>70% of correct responses). In this regard, our results agree with those of previous studies that have observed that FSD is little known (3, 33), silenced, or normalized (37, 38). Although information related to incontinency has been increasingly disseminated in recent years (18), information related to sexual function remains lacking (37–39). Access to sexual health information and care is often very limited (39), especially for females in menopausal stages (40). As dyspareunia and other FSD symptomatology remain common and normalized among females (3), more educational approaches are needed to address this public health issue.

With reference to knowledge related to the rest of the PFD, only athletes who attended the entire educational program reached the appropriate level of knowledge in all PFD domains. Athletes who partially attended the educational program also improved the UI domain. These results were anticipated because the content of the educational program was distributed throughout all three sessions. Moreover, the program frequently addressed content related to UI because it is one of the most frequent PFD among female athletes, and a supportive environment is required to manage and prevent it (41, 42).

These improvements were observed even for domains in which the athletes had already reached an appropriate level of knowledge. In contrast, a recent study did not observe any significant increase in UI knowledge after a 90-min educational session, suggesting that it may be difficult to improve knowledge in domains where athletes already have an appropriate level (3). Another study proposed a 90-min workshop that increased knowledge levels; however, it had to be complemented by a 60-min theoretical seminar (18). Because we observed improvements even in athletes with previous knowledge only after they completed 180 min of the educational program (three sessions of 60 min), the program's insufficient duration should be considered a crucial aspect in designing educational strategies.

It is also remarkable that participants in this study was highly educated female athletes (86.9% university education), with a large proportion having backgrounds in health-related fields (68.5% in health-related fields). It is important to note that, to our knowledge, there are no publicly available national statistics detailing the educational level of females participating in sports. While this may have facilitated engagement with the educational content, this profile may not reflect the broader population of female athletes, particularly those with lower educational attainment or from non-health-related education disciplines.

Only athletes who attended the educational program showed reduced unhealthy habits during micturition, with a lower number of these habits, than athletes who did not attend the educational program. Although almost all these unhealthy habits were reduced among full-attendance athletes, the two most reduced habits after the intervention were straining and voiding to completely empty the bladder to initiate urination. Before the program, 21.4% and 15.7% of full-attendance athletes had these habits, which were corrected to 12.9% and 7.1% 4 weeks after the intervention. However, more than 45.0% of full-attendance athletes continued to have habits such as voiding without desire at home despite the program. This habit was even more frequent among partial-attendance and no-attendance athletes, at 56.5% and 64.9%, respectively. Similar outcomes were reported in a study that designed PF educational sessions specifically for female teenagers (14). These authors reported that >90% of the girls corrected their urinary habits, whereas >50% improved their defecation habits. The reduction of these detrimental practices is crucial because of their relationship with PF function deterioration (43). Straining for voiding induces descent of the bladder neck and puborectalis muscle, leading to a stressful situation for the PF (43).

Similarly, unhealthy habits during defecation were reduced only by full-attendance athletes. We should highlight the significant correction of the habit of waiting until coming home to defaecate and pushing down to initiate defecation. Before the program, these habits were sustained by 37.1% and 32.9% of full-attendance athletes. After the program, 21.4% and 22.1% of the athletes had these habits. However, we found that 61.4% of the full-attendance athletes had frequent habits, such as trying to defaecate at home, despite the educational program. Among partial-attendance and non-attendance athletes, the percentages were 60.9% and 73.0%, respectively. As the process of stool elimination involves complex coordination of the abdominal and pelvic musculature and the anal sphincter, it needs to be activated when the rectum is distended by the presence of fecal content (44). Premature or “just in case” defecation could lead to PF disorders owing to the extra effort that females must exert to empty the rectum without sufficient distension.

Very few studies have explored unhealthy habits during sports practice, despite the importance of avoiding certain exercises to care for PF structures (12). In our study, although we did not observe any significant reduction compared to baseline in any group, after the intervention period, athletes who attended the complete educational program had fewer unhealthy habits during their sports practice than athletes who did not attend any educational session. In general, the most frequent practice among participants was forgetting to voluntarily activate the PF musculature just before and/or during efforts that increased the intra-abdominal pressure. This unhealthy habit was very frequent in all athletes, being performed by 40.0% of full-attendance athletes, 52.2% of partial-attendance athletes, and 62.2% of non-attendance athletes, even after the program. Previous studies have demonstrated that correct voluntary preparative contraction of the PF muscles, also known as the “the knack principle,” can reduce or prevent urine leakage. During this correct contraction, the PF musculature lifts inwards, the urethra closes, and the PF musculature stabilize the urethra (45, 46). However, it is important to teach athletes this technique because many are unable to perform the correct PF muscle contraction. Instead, they often engage superficial muscles or strain unnecessarily (47). For this reason, contractions are more effective when athletes are aware of and practice the correct musculature contractions (12).

Consequently, in our educational program, we added 3D anatomical models and practical exercises to help athletes become conscious of their PF structures and to facilitate proper contractions. Although we cannot confirm whether these contents improved PF contractions, the utility perceived by the athletes was very high. This is a crucial aspect of engaging participants, especially in programs composed of several sessions. In line with this, a recent study showed a high level of satisfaction and participation by females in PF education workshops in a web-based conference format (14). These results confirm the significant interest in this topic among the female population. We should note that our educational proposal included content adapted to the daily demands of athletes, with frequent examples and situations regarding the sports training.

It is important to note that, in this study, changes in habits related to PF care were assessed by the reduction in unhealthy habits as a numerical count of dichotomized behaviors. This approach was chosen to facilitate clarity and feasibility in data collection and analysis, particularly within a sporting population where time and attention are limited. However, it may oversimplify the complexity of behavioral change because dichotomization can obscure variations in frequency, intensity, and context, which are important dimensions of habit formation and maintenance. We should highlight that any observed changes in habits may be interpreted as improvements in awareness and self-monitoring rather than direct behavioral modifications. This distinction is important, as self-reported data may reflect increased recognition of previously unnoticed behaviors rather than actual habit change. Future studies should incorporate more nuanced metrics, such as frequency, intensity, or contextual factors, to better reflect meaningful and sustained behavioral change.

Although the educational program was highly valued by participants, with one-third of athletes giving it the highest rating, the observed reduction in unhealthy habits was limited. This suggests that behavioral change may require more time and reinforcement than a short-term intervention can offer.

Our study had certain limitations. First, as the participants were a female sport population, with a high proportion of university-educated participants, our results cannot be extrapolated to other females. Second, our results are based on self-reported questionnaires. To avoid misunderstandings during information collection, the questionnaire was answered by 12 female athletes and reviewed by six external experts. All participants confirmed their understanding of every question and the time they needed to respond. Although questionnaires are appropriate for exploring PFD symptoms, their inherent limitations include the potential influence of social desirability bias. Participants may report behaviors they perceive as socially acceptable rather than their actual practices. This bias may lead to overestimation of positive outcomes, particularly in interventions addressing sensitive topics such as pelvic floor health (48). To reduce it and reinforce the anonymity and confidentiality, the following statement was incorporated in the explanation of the questionnaire: *We kindly ask you to respond as openly and honestly as possible. Your individual experience—whether typical or not—is valuable and will contribute meaningfully to the quality and relevance of this research. All responses are anonymous and treated with strict confidentiality*. Additionally, future studies should consider incorporating complementary methods such as clinical evaluations, digital tracking tools to enhance the objectivity and reliability of behavioral outcomes, or even social desirability scales (49). Also, our study design was compounded by three non-equivalent groups, which could be biased owing to the interests of the participants. In this regard, we preferred to collect final data from athletes who did not attend the program as the control group, despite anticipating a low number of responses 4 weeks later. This design allowed for a comparison of different levels of participation. However, the use of a non-randomized control group, composed of participants who did not attend the intervention, introduces a self-selection bias that limits the internal validity of the study. As participation was voluntary, it is likely that individuals with greater interest or concern regarding pelvic floor health were more inclined to engage with the program, potentially influencing the observed outcomes. To mitigate this, the training was titled *Understanding and Caring for Your Pelvic Floor: An Educational Program for All Female Athletes* and was accompanied by inclusive messaging explicitly stating that the program was intended for all females, regardless of whether they experienced pelvic floor dysfunction. Future studies should consider randomized controlled designs or matched comparison groups to strengthen internal validity and reduce selection bias. Because 4 weeks may be insufficient to evaluate long-term behavior changes, we recommend future studies with longer follow-up periods to assess habit retention and potential relapse.

Educational programs on PF are needed to improve the knowledge and habits regarding its care among female athletes. The inclusion of innovative resources in the design may ensure the perception of athletes' utility. This aspect may be especially relevant in the case of multiple-session programs because engagement ensures attendance in the entire educational program and thus improves knowledge and habits.

As a conclusion, a three-session innovative online educational program on PF is useful for improving knowledge about PF disorders and habits related to its care in female sports populations. Full attendance of an educational program is crucial for the acquisition of an appropriate level of knowledge and habits.

## Data Availability

The datasets presented in this study can be found in online repositories. The names of the repository/repositories and accession number(s) can be found below: Research Data Repository (RDR) of the Collective Catalog of the Universities of Cataluña (CSUC) through the agreement established with the University of the Balearic Islands (UIB): https://doi.org/10.34810/data1833, CORA. Repositori de Dades de Recerca, V1.
