# Supplementary material for: Effects of an innovative educational program on female athletes' knowledge and habits related to pelvic floor care: a quasi-experimental study
Source: Front Public Health. 2025 Sep 5;13:1664696. doi: 10.3389/fpubh.2025.1664696 (PMC12446254; doi:10.3389/fpubh.2025.1664696)
Supplement: Supplementary file 1 [file Table_1.pdf]

Supplementary table 1. Unhealthy habits among participants before and after the intervention period.

SPORTS TRAINING UNHEALTHY HABITS		%NA athletes with this habit		%PA athletes with this habit		%FA athletes with this habit	
		Pre	Post	Pre	Post	Pre	Post
#1	Cuando entreno o compito. noto un descenso hacia abajo (hacia los pies) de mi suelo pélvico.	0.0	2.7	0.0	4.3	5.7	2.9
#2	Evito consultar con un profesional sanitario (médico. fisioterapeuta. o cualquier otro) con relación a aspectos de mi entrenamiento que pueden afectar a mi salud pélvica.	13.5	8.1	8.7	13.0	7.1	2.9
#3	Cuando realizo esfuerzos importantes durante mi entrenamiento. mi abdomen protruye hacia afuera (se abomba).	5.4	5.4	8.7	13.0	2.9	5.7
#4	Evito el uso de dispositivos perineales (i.e. paraguas vaginales) durante mis entrenamientos o competiciones. independientemente del grado de esfuerzo que tenga que realizar.	13.5	21.6	4.3	21.7	15.7	17.1
#5	Al coger un objeto o peso del suelo. acostumbro a encorvar la columna hacia delante.	21.6	21.6	17.4	17.4	15.7	17.1
#6	Evito consultar con profesionales sanitarios (médico. fisioterapeuta o cualquier otro). cuando tengo algún problema o síntoma relacionado con mi suelo pélvico (i.e. pérdida de orina. sensación de bulto o peso en el interior de mi pelvis. dolor o molestias durante mis actividades sexuales. etc).	8.1	8.1	4.3	4.3	7.1	5.7
#7	Cuando entreno o compito y tengo que realizar esfuerzos de carga importantes (i.e. ejercicios de levantamiento de peso). suelo aguantar la respiración (es decir. me mantengo en apnea).	21.6	24.3	17.4	30.4	14.3	15.7
#8	Cuando entreno. mantengo la intensidad o el volumen de entrenamiento a pesar de que note cualquier síntoma leve en mi suelo pélvico (i.e. pérdida de orina. gas o abultamiento. pesadez o presión).	27.0	32.4	8.7	13.0	10.0	10.0
#9	Olvido activar de forma voluntaria mi suelo pélvico cuando realizo un esfuerzo que supone carga de peso (i.e. ejercicios de pesas) o en la recepción tras un impacto (i.e. ejercicios de saltos).	62.2	62.2	47.8	52.2	25.7	40.0
MICTURITION UNHEALTHY HABITS							
#1	Trato de vaciar mi vejiga en mi casa.	51.4	64.9	60.9	56.5	50.0	45.7

#2	Intento esperar a vaciar mi vejiga hasta que llego a casa.	21.6	29.7	21.7	8.7	17.1	10.0
#3	Vacío mi vejiga sin sentir necesidad de orinar. antes de entrenar.	35.1	48.6	39.1	39.1	24.3	22.9
#4	Intento retrasar el vaciamiento de la vejiga si estoy ocupada.	27.0	29.7	30.4	34.8	21.4	25.7
#5	Espero a vaciar mi vejiga hasta que ya no puedo contener la orina.	18.9	13.5	4.3	4.3	12.9	10.0
#6	Refreno el deseo de orinar mientras estoy en el trabajo.	18.9	16.2	4.3	13.0	18.6	12.9
#7	Empujo hacia abajo para iniciar el proceso de orinar.	21.6	29.7	26.1	13.0	15.7	7.1
#8	Empujo hacia abajo para que la orina fluya durante el proceso de orinar.	21.6	29.7	26.1	17.4	12.9	8.6
#9	Empujo hacia abajo para vaciar la vejiga completamente.	45.9	45.9	47.8	34.8	21.4	12.9
#10	Me agacho (floto) sobre el inodoro, sin apoyarme en la taza, cuando vacío mi vejiga.	10.8	8.1	8.7	0.0	7.1	7.1
DEFECATION UNHEALTHY HABITS							
#1	Trato de defecar en mi casa.	78.4	73.0	56.5	60.9	67.1	61.4
#2	Intento esperar a defecar hasta que llego a casa.	51.4	56.8	26.1	34.8	37.1	21.4
#3	Intento defecar antes de ir a entrenar. aunque no tenga ganas.	8.1	10.8	4.3	13.0	2.9	5.7
#4	Defeco sin sentir necesidad, en casa.	0.0	2.7	4.3	8.7	2.9	2.9
#5	Intento retrasar la defecación si estoy ocupada.	29.7	24.3	4.3	17.4	10.0	15.7
#6	Espero a defecar hasta que ya no puedo contenerme.	10.8	10.8	4.3	17.4	4.3	7.1
#7	Refreno el deseo de defecar mientras estoy entrenando.	18.9	21.6	4.3	13.0	11.4	8.6
#8	Mantengo la respiración (apnea) y empujo para poder iniciar o hacer progresar la salida de las heces.	27.0	27.0	26.1	26.1	20.0	15.7
#9	Empujo hacia abajo para iniciar el proceso de defecación.	27.0	32.4	21.7	34.8	32.9	22.9
#10	Me agacho (floto) sobre el inodoro. sin apoyarme en la taza. cuando defeco.	0.0	2.7	0.0	0.0	1.4	2.9

FA, full-attendance athletes; NA, non-attendance athletes; PA, partial-attendance athletes.
